# Psychiatric Disorders and Polyphenols: Can They Be Helpful in Therapy?

**DOI:** 10.1155/2015/248529

**Published:** 2015-06-09

**Authors:** Jana Trebatická, Zdeňka Ďuračková

**Affiliations:** ^1^Department of Child and Adolescent Psychiatry, Faculty of Medicine, Comenius University and Child University Hospital, 833 40 Bratislava, Slovakia; ^2^Institute of Medical Chemistry, Biochemistry and Clinical Biochemistry, Faculty of Medicine, Comenius University, 813 72 Bratislava, Slovakia

## Abstract

The prevalence of psychiatric disorders permanently increases. Polyphenolic compounds can be involved in modulation of mental health including brain plasticity, behaviour, mood, depression, and cognition. In addition to their antioxidant ability other biomodulating properties have been observed. In the pathogenesis of depression disturbance in neurotransmitters, increased inflammatory processes, defects in neurogenesis and synaptic plasticity, mitochondrial dysfunction, and redox imbalance are observed.* Ginkgo biloba*, green tea, and* Quercus robur* extracts and curcumin can affect neuronal system in depressive patients. ADHD patients treated with antipsychotic drugs, especially stimulants, report significant adverse effects; therefore, an alternative treatment is searched for. An extract from* Ginkgo biloba *and from* Pinus pinaster *bark, Pycnogenol, could become promising complementary supplements in ADHD treatment. Schizophrenia is a devastating mental disorder, with oxidative stress involved in its pathophysiology. The direct interference of polyphenols with schizophrenia pathophysiology has not been reported yet. However, increased oxidative stress caused by haloperidol was inhibited* ex vivo* by different polyphenols. Curcumin, extract from green tea and from* Ginkgo biloba, *may have benefits on serious side effects associated with administration of neuroleptics to patients suffering from schizophrenia. Polyphenols in the diet have the potential to become medicaments in the field of mental health after a thorough study of their mechanism of action.

## 1. Introduction

The prevalence of psychiatric disorders permanently increases. In the WHO European Region nearly 4 out of 15 people suffer from major depression and anxiety. Neuropsychiatric disorders are the second cause of disability in Europe and account for 19% in comparison to cardiovascular disorders with only 4%. In 28 countries of EU with a population of 466 million, at least 21 million people were affected by depression, out of which almost 80% are men. The treatment of psychiatric disorders is very expensive. The total annual cost of depression in Europe was estimated at Euro 118 billion in 2004, which corresponds to a cost of Euro 253 per inhabitant. The cost of depression corresponds to 1% of the total economy of Europe. These reasons provide support for the importance of increased research efforts in this field, better detection, prevention based on improvement of life-style factors including diet, and effectiveness of treatment [1].

Results of many animal and human studies support the role of different natural polyphenolic compounds in modulation of mental health including brain plasticity, behaviour, mood through anxiolytic, antidepressant-like properties, and cognition. Research demonstrates that dietary factors and exercise can affect the maintenance and development of neurons and protect the brain from insult associated with neurological illnesses or injuries [2–7].

## 2. Polyphenolic Compounds

**Figure 1:**
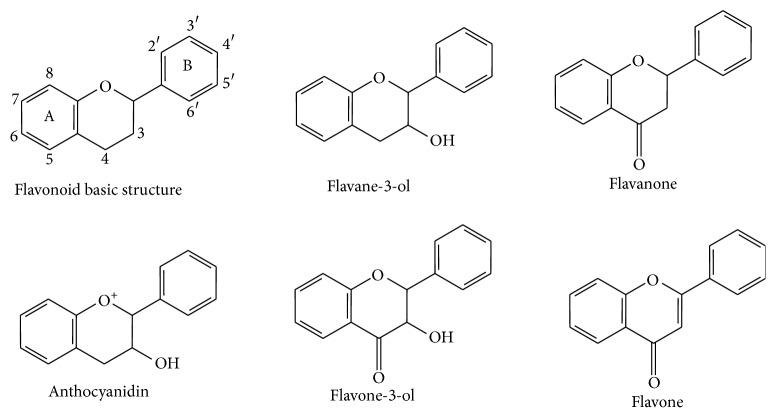
Basic flavonoid structures.

Polyphenols (known as* polyhydroxyphenols*) are predominantly secondary metabolites of plants. They belong to structural class of organic compounds characterized by the presence of large multiples of phenol structural units. These phenol structures underlie the unique physical, chemical, and biological (metabolic, toxic, therapeutic, etc.) properties of particular members of the class. The name derives from the ancient Greek word *πολύς* (polus, meaning “many, much") and the word phenol which refers to a chemical structure formed by attaching a hydroxyl (–OH) group to an aromatic phenyl ring. They are divided into three groups according to their hydrolytic cleavage products: (i) tannins: derivatives of catechin or gallic acid with mostly antioxidant properties [8], (ii) phenylpropane derivatives (lignans, ellagitannins, cinnamic acid derivatives, and others); for example, higher dietary intake of lignans is associated with better cognitive functions in postmenopausal women [9] or extract of ellagitannins from oak wood reduced many of key symptoms of chronic fatigue [10], and (iii) flavonoids: phenolic compounds spread in the plant kingdom. They include more than 4000 different derivatives and their list constantly increases. Formation of so many derivatives is possible due to the substitution of hydrogen atoms by hydroxyl, methoxyl, and other groups at different sites of the basic structures. The basic flavonoid structures include the following:* flavan-3-ols* (epicatechin and gallocatechin),* flavanones* (naringenin and hesperidin),* flavones* (apigenin and luteolin),* flavone-3-ol* (quercetin and myricetin),* anthocyanidins* (cyanidin and pelargonidin), and* isoflavones* (genistein and daidzein) [11] (Figure 1).

Polyphenols occur in food (vegetables and fruits) either as free monomers (quercetin and catechin) or oligomers (procyanidins). They are bound to saccharides as glycosides or occasionally they are found as free aglycones. After ingestion, flavonoids can undergo biotransformation to their metabolites which can be detected in plasma reaching concentration of about 1 *μ*mol·L^−1^ [12, 13].

Consumption of polyphenol-rich foods is associated with a lower incidence of coronary heart disease, myocardial infarction [14], cancer [15], neurodegenerative diseases [16], psychiatric disorders (like ADHD) [17], and other chronic diseases [18]. Since in the pathology of these diseases, in addition to other factors, oxidative stress has been assumed to play a role, dietary flavonoids have been suggested to exert health benefits through antioxidant mechanisms. In experiments* in vitro*, flavonoids exert a significant antioxidant [8, 19] and redox modulating [20] ability. Polyphenols act as strong antioxidants* in vitro* through the numerous mechanisms, such as radical scavenging, metal ions (Fe, Cu, and others) chelation, and the modulation of antioxidant enzyme activities [8, 21]. In the scavenging ability the position and the number of phenolic –OH groups play a role through donation of a hydrogen atom from their hydroxyl groups to radicals, resulting in radical moiety elimination. During this reaction, phenoxyl radical is formed that can form stable compound and terminates radical reaction via reaction with another radical [20].

Upon consumption of food, polyphenols are available in the form of esters, glycosides, or polymers that cannot be absorbed in the intestine. The original molecules of polyphenolic compounds are hydrolyzed by microbial enzymes in colon and transformed via methylation, sulfation, and glucuronidation to derivatives of original molecules followed by their absorption in the colon and travelling through blood to various tissues and organs such as the brain. However, due to the diverse susceptibilities of phenolic compounds to colon enzyme metabolism, their bioavailability can vary from very low to very high [22, 23]. The low bioavailability and transformation of polyphenolic compounds* in vivo* to different derivatives lead to their low direct antioxidant activity in comparison to other low-molecular weight antioxidants, such as vitamins C and E and uric acid [24].

However, in addition to antioxidant activities polyphenols at low concentration can exert also other biological effects* in vivo*. Polyphenols can participate in modulation of different signaling pathways, thus influencing the fate of cells [25] including nerve cells via influencing the neuronal survival, regeneration, development, or death [26]. Polyphenolic compounds possess also antimutagenic ability [27], vasodilating [28], antithrombic [29], antiapoptotic [30], and anti-inflammatory [31] effects.

Anti-inflammatory effects of polyphenols in cerebral tissue can be realised via binding to various receptors. Flavonoid-induced receptor stimulation can modulate active state of different kinases, such as the mitogen-activated protein (MAP) kinase (naringenin), phosphoinositide-3-(PI3) kinase (curcumin), nuclear factor-kappaB (resveratrol and epigallocatechin gallate), and protein kinase C (PKC) pathways (resveratrol). Polyphenols can thus influence differentiation and apoptosis, cell survival (inhibition of apoptosis), inflammatory response, learning and memory, and reduction of amyloid plaque formation. Polyphenols can affect the activation of glial cells in brain, which are the residence of macrophages, via inhibiting the inflammation mediated by macrophages through the reduction of proinflammatory cytokines (IL-1 beta, TNF-alpha) formation [32].

The neuroprotection of polyphenols can be assigned to the improvement of cerebral blood flow via stimulation of NO formation in the endothelium and inhibition of platelet aggregation [32].

The principle question is how and in which form and amount can polyphenols reach the brain and modulate its function? This problem is not completely solved. Results from animal experiments indicate that diet supplementation with some polyphenolic extracts (e.g., from grapes, blueberries, and blackberries) results in deposition and bioavailability of polyphenols and their metabolites in the brain of animals where they can directly exert their protective effects. However, we can only assume that a diet rich in vegetables and fruits will result in increased cerebral deposition of these beneficial compounds. Polyphenols might modify brain function at three locations: (i) outside the CNS, by improving cerebral blood flow or by altering signaling pathways from peripheral organs to the brain, (ii) at the blood-brain barrier, by altering multi-drug-resistant protein-development influx/exflux mechanisms of different biomolecules, and (iii) inside the CNS, by modifying the activity of neurons and glial cells [33].

On the other hand, flavonoids could exert also their prooxidative properties* in vitro*, for example, in the presence of high concentration of Cu^2+^ ions (25–100 *μ*mol·L^−1^) and oxygen. The issue whether prooxidative effects of flavonoids can be exerted also* in vivo* has not been addressed yet and the answer to this question requires further studies [34, 35].

## 3. Psychiatric Disorders

Psychiatric disorders, including major depression, attention deficit hyperactivity disease (ADHD), and schizophrenia, contribute largely to mental problems of children, adolescents, and adults.

According to the PubMed, under the terms “polyphenols” and “mental health,” the number of studies dealing with polyphenols and mental health is much lower (21 papers) compared with cardiovascular diseases (924 papers) or neurodegenerative diseases (291 papers). For this reason, any new paper dealing with this topic is very important.

**Figure 2:**
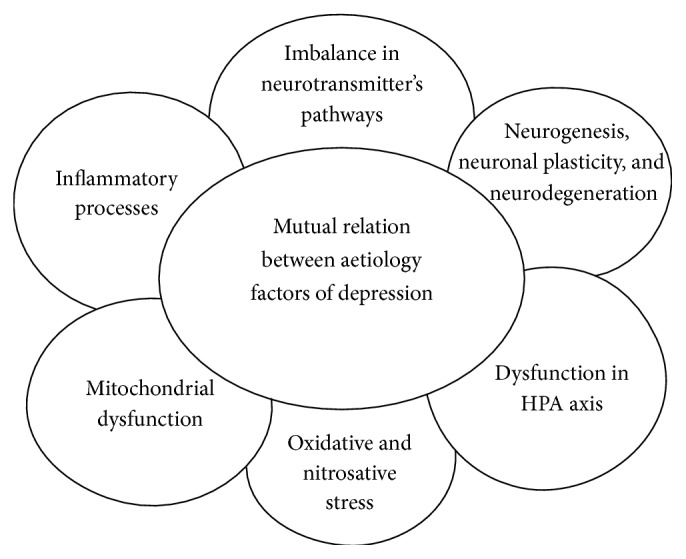
Mutual relations between aetiology factors of depression. Explanation of individual factors is given in the text. HPA: hypothalamic-pituitary-adrenal.

It is believed that in pathology of psychiatric disorders also oxidative stress plays a role (Figure 2). Oxidative stress is defined as the disbalance between production of free radicals and/or reactive oxygen species/reactive nitrogen species (ROS/RNS) and antioxidant defence in favour of ROS/RNS leading to oxidative damage to lipids, proteins, and DNA and thus to the dysfunction of cells and organs [25]. Although the brain forms less than 2% of the body weight, it consumes about 20% of the oxygen available through respiration. The brain is also a lipid-rich organ, which can contribute to its susceptibility to oxidative damage [36]. The brain has a large potential oxidative capacity but a limited ability to counteract oxidative stress. Cells in the central nervous system are more sensitive to toxic effects of ROS, than cells in other organs of the body. Moreover, in the brain there is a low activity of catalase, insufficient activities of glutathione peroxidase (both enzymes decompose hydrogen peroxide or organic peroxides) and superoxide dismutase (decomposes superoxide anion radical to hydrogen peroxide and oxygen), and higher level of iron ions and ascorbic acid (together they form optimal conditions for formation of very toxic hydroxyl radical) in comparison to other cells. These facts increase the susceptibility of brain to oxidative and peroxidative damages to biomolecules [25, 32].

In addition to antioxidant properties of polyphenols, research has shown that polyphenols can exert their neuroprotective properties through modulation of specific cellular signaling pathways involved in cognitive processes such as synaptic plasticity, notably, pathways with CREB (cAMP-response element-binding protein) signalling. CREB is a transcription factor linked with genes that express brain-derived neurotrophic factor (BDNF). The importance of CREB in brain function is emphasized by studies that demonstrate impairments in memory formation induced by the disruption of CREB activity and, similarly, accelerations in memory formation stimulated by increased CREB activity [37]. Polyphenols can directly modulate these signaling pathways by induction of CREB and subsequently by BDNF activation [23].

## 4. Major Depression

Major depression is a psychiatric disorder which represents the fourth leading cause of disability worldwide and is expected to become the second most prevalent disease after ischemic heart disease by 2020. Depression is also one of the most costly disorders in western countries, and antidepressants account for 20% of total CNS drug sales [38].

Depression has a multifactorial aetiology arising from genetic, environmental, psychological, and biological factors. These factors are mainly applied jointly in aetiology of depression, and their impact on the status and severity of disease are mutually intertwined (Figure 2).

**Figure 3:**
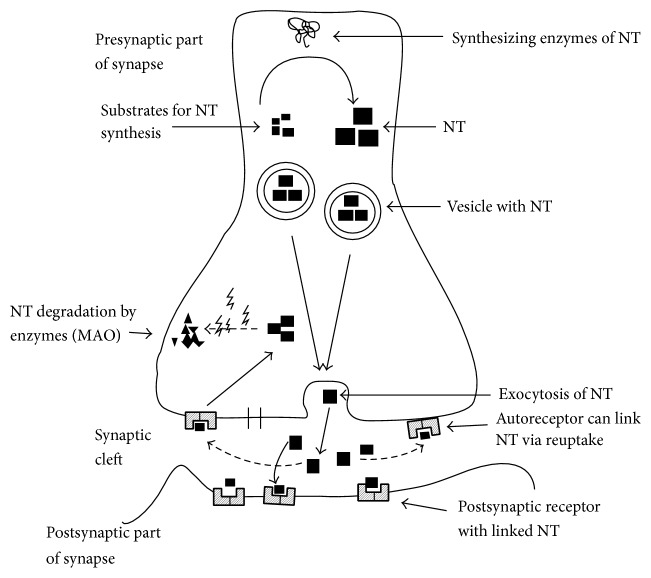
Neurotransmitters in synapse. Molecules of NT are synthesized from their substrates by enzymes. NT are stored in vesicles. Vesicles after action potential fuse with presynaptic membrane and NT are released into synapse cleft. Released NT are linked to postsynaptic receptors and signal is transferred (→) to postsynapse. NT can be reuptaken by autoreceptor and neurotransmission is inhibited (⇢). Reuptaken NT can be enzymatically degraded (MAO). NT: neurotransmitter; MAO: monoaminooxidase.

*Firstly*, the causal relation is assumed between disturbance in monoamine (neurotransmitters) metabolism, especially serotonin, abnormalities in its receptor, and depression [39], but also dopamine, adrenaline, and glutamate are involved [40, 41]. Metabolism of neurotransmitters is influenced by enzymes involved in their degradation, like monoamine oxidase (MAO), and in synthesis of their amino acid precursor tryptophan by enzyme tryptophan hydroxylase [42]. The causal association between neurotransmitters and depression is also based on successful treatment of depression with selective serotonin reuptake inhibitors (SSRIs) into the presynaptic cells increasing the level of serotonin in the synaptic cleft available to bind to the postsynaptic receptor (Figure 3). The process of neurotransmission is explained in the text to the figure.

*Secondly*, increased inflammatory processes are also involved in the pathology of depression which was first reported by Maes et al. [43]. Increased proinflammatory cytokines can induce various clinical characteristics such as disturbed serotonin metabolic pathway and neurovegetative symptoms [44]. Increased level of proinflammatory cytokines (IL-6, tumor necrosis factor (TNF-alpha)) and C-reactive protein (CRP) in blood are recognized as good markers of increased inflammation in depressive patients. These reliable markers of nonspecific inflammation showed positive correlation with the severity of depressive symptoms and some comorbidities like impaired sleep, cognitive dysfunction, and fatigue [38, 45, 46].

*Thirdly*, neurogenesis and synaptic plasticity in the context of adult hippocampal neurogenesis (AHN) are compromised in patients with depression with subsequent neurodegeneration [47]. This results in stress-induced alteration in the number and shape of neurons and glial cells in brain regions of depressed patients and decreased proliferation of neural stern cells [48]. The most abundant neurotrophin in the central nervous system involved in neuronal survival, growth, and proliferation is the brain-derived neurotrophic factor (BDNF). In patients with depression the levels of BDNF are very low [49, 50].

*Fourthly*, dysfunction in hypothalamic-pituitary-adrenal (HPA) axis is characteristic for patients suffering from depression [51]. This gives rise to the failure in the secretion of cortisol and glucocorticoids depending on the type of depression and to the hypersecretion of corticotropin-releasing hormone (CRH). Treatment with antidepressants regulates levels of these hormones [52–54].

*Fifthly*, depression is associated with mitochondrial dysfunction related to lower activities of respiratory chain enzymes, ATP production, and damage to mitochondrial DNA [55–58].

*Sixthly*, it is assumed that redox imbalance (increased oxidative and nitrosative stress) also plays a role in the pathology of depression. The antioxidant defence systems are decreased and the level of low molecular-weight antioxidants, such as vitamin C, vitamin E, and coenzyme Q as well as the activity of antioxidant enzyme glutathione peroxidase are reduced [59–62]. Deficiencies in antioxidant defence systems impair protection of cells and organs against free radicals and reactive oxygen and nitrogen species leading to the damage to proteins, DNA, and lipids through oxidation of fatty acids in phospholipids of lipoproteins and membranes [8].

Increased oxidative stress is manifested by increased markers of oxidative stress in depressed patients, such as increased level of malondialdehyde and isoprostanes, products of lipoperoxidation [63, 64], peroxides in plasma [65], xanthine oxidase activity [66], and oxidative damage to DNA represented by increased level of 8-hydroxy-2-deoxyguanosine [67, 68].

### 4.1. Can Polyphenols Influence Aetiology Factors of Depression?

Several* in vitro* and* in vivo* studies indicate that polyphenols can affect neuronal system and processes [3]. It should be kept in mind that the effects of polyphenols* in vivo* may be different from the effects* in vitro*, as* in vivo* rather metabolites of polyphenols are active than original substances. For example, curcumin, a nonflavonoid phenolic compound present in* Curcuma longa*, known and used in Indian traditional medicine, after administration significantly decreased depression-like behaviour in rats probably through improvement of the BDNF level [69]. Curcumin coadministered with alkaloid piperine inhibited MAO activity and increased serotonin and dopamine level in mice [70]. Sanmukhani et al. [71] provides clinical evidence that curcumin (1000 mg/day) administered for 6 weeks to 60 patients with major depression in randomized and placebo controlled trial may be used as an effective and safe modality for treatment of depressive patients without concurrent suicidal ideation. On the contrary to results of Sanmukhani et al., Bergman et al. [72] did not observe significant differences between the groups of patients with administered curcumin (500 mg) and placebo for 5 weeks in randomized, double-blind, and placebo controlled clinical study, probably due to low daily doses used. However, the patients in the curcumin group demonstrated a trend to a more rapid relief of depressive symptoms in comparison to those in the placebo group.

Curcumin is a lipophilic compound that can easily cross the blood-brain barrier and directly induce neuroprotection probably through its antioxidant ability to inhibit lipid peroxidation and neutralize ROS and RNS [73]. In addition, curcumin can affect number of cellular pathways on molecular level and via anti-inflammatory properties it can inhibit cyclooxygenase 1 and cyclooxygenase 2 and influence many other signalling pathways leading to cell protection and enhancement of cell survival [74].

Flavonoid derived from catechin, epigallocatechin gallate (EGCG), present in green tea, was used in traditional Chinese medicine for at least 4000 years. At present EGCG is known for its powerful antioxidant properties and for its ability to attenuate stress and depression. In experimental study on mice increased level of BDNF was found after a long-term administration of green tea polyphenols [75] or reduced serum corticosterone and adrenocorticotropic hormone levels after forced swimming test [76].* In vitro* experiments with cultured hippocampal neurons confirmed the specific modulation of the GABA-A receptor benzodiazepine site by application of EGCG [77]. In a double-blind, randomized, and placebo controlled human study with seventy-four subjects who were administered green tea or placebo for 5 weeks, subjects with the long-term green tea extract supplementation increased the reward learning and prevented the depressive symptoms [78]. Also an extract of polyphenols from* Ginkgo biloba,* consisting, namely, of flavonol quercetin and kaemferol, has been shown to have antidepressant-like effects in mice probably through increasing BDNF level, neuronal survival and plasticity, and inhibition of MAO towards serotonin [79]. MAO is also inhibited* in vitro* by anthocyanins from berries, flavone apigenin from celery, and stilbene* trans*-resveratrol from red wine [80]. The flavonoids from cocoa showed also antidepressant-like effects in the animal model using the forced swimming test in rats [81] and reduced symptoms of chronic fatigue in ten subjects enrolled in double-blind, randomized, clinical pilot crossover study [82]. Polyphenolic extract from the wood of* Quercus robur* (*Robuvit*, Horphag Res. Ltd.) is a proprietary extract with concentrated water-soluble components of the wood (ellagitannins) also found in wine resting in oak barrels. Robuvit contains roburins (A, B, C, D, and E) and grandinin. These components belong to the group of hydrolysable tannins (ellagitannins). Clinical investigation in healthy volunteers and patients with primary lymphedema has shown an increased antioxidant capacity of blood and a decrease in peripheral edema after supplementation with Robuvit [83, 84].

In the study of Natella et al. [83], Robuvit actions were studied on modulation of gene expression. Robuvit affects ribosomes, cell cycle, and spliceosome pathway. The effects of Robuvit on stimulation of ribosomal activity and protein translation are suggested to be involved in relieving fatigue in healthy volunteers and chronic fatigue syndrome in patients [85]. In addition, Robuvit was shown to accelerate healing in patients with temporary hepatic damage [86]. In another study, intake of 300 mg/day of Robuvit was associated with improving effect on energy, tiredness, and tension subscales evaluating fatigue in 20 volunteers with lower baseline of feelings scoring [87]. In the same volunteers, the decrease of markers of oxidative stress and increase of activity of antioxidant enzymes, Cu/Zn superoxide dismutase, catalase, and total antioxidant capacity of plasma* in vivo* were observed [88].

## 5. Attention Deficit Hyperactivity Disorders (ADHD)

Attention deficit hyperactivity disorder is the most prevalent childhood disorder, estimated to affect 2–18% of children depending largely on diagnostic criteria [89]. The economic consequences of ADHD persisting into adulthood are significant with one US analysis finding an average of 35 days of annual lost work performance, representing 120 million days of annual lost work in the labor force, equivalent to 19.5 billion USD lost human capital [90].

ADHD is a complex polygenic disorder with high levels of heterogeneity, influenced by the interaction of multiple aetiological factors [91]. Twin, family, and adoption studies of ADHD have supported a strong genetic contribution to the disorder, with heritability ranging from 60 to 90%. A plausible genetic hypothesis for ADHD is a mixture of dominant and recessive major genes that act with complex polygenic transmission patterns. Molecular genetic studies have implicated a number of possible genes (DRD4, DRD5, DAT1, DRD1, and Taq1). However, each of these genes only increases relative risk of ADHD slightly. Pre-, peri-, and postnatal environmental factors play an important role in the pathogenesis of ADHD. Prenatal factors are associated with maternal lifestyle during pregnancy. For example, prenatal alcohol exposure is known to induce brain structural anomalies, especially in the cerebellum. Maternal smoking produces a 2.7-fold increased risk for ADHD. Perinatal factors have also been implicated, with a twofold increase in ADHD in very low-birthweight children and an increased rate of pregnancy with birth complications. Among postnatal factors, a role for malnutrition and dietary deficiency in ADHD has been proposed. An imbalance of essential fatty acid (omega-3 and omega-6) intake has been suggested to be potentially involved in the development of ADHD. Iron deficiency has been implicated in some cases. Early deprivation of social environment during the postnatal period may also have significant effects [92].

Studies have identified various structural and functional abnormalities in frontostriatal network. This network involves the lateral prefrontal cortex, the dorsal anterior cingulate cortex, and the caudate nucleus and putamen. In ADHD patients, reductions in volume have been observed in total cerebral, the prefrontal cortex, the basal ganglia (striatum), the dorsal anterior cingulate cortex, the corpus callosum, and the cerebellum. A developmental trajectories study in ADHD patients showed a delay in cortical maturation. The delay was most prominent in prefrontal regions important in the control of cognitive processes including attention and motor planning. Compensatory networks including basal ganglia, insula, and cerebellum have been implicated for relative lower cognitive load tasks in ADHD patients.

Genetic influences predispose a child to catecholaminergic dysregulation (deficits in dopamine, noradrenaline, and serotonin transmission) and abnormalities in their metabolism [93, 94]. There is also persuasive relation between ADHD and suboptimal level of catecholamines and the composition of consumed essential fatty acids [95] as well as consumption of certain additives or food preservatives [96].

For diagnosis clear evidence of clinically significant impairment in social, academic, or occupational functioning is required. The essential feature of ADHD is a persistent pattern of inattention and/or hyperactivity-impulsivity that interferes with functioning or development. Inattention manifests behaviorally in ADHD as wandering off task, lacking persistence, having difficulty sustaining focus, and being disorganized which is not due to defiance or lack of comprehension. Hyperactivity refers to excessive motor activity (such as a child running around) when it is not appropriate, or excessive fidgeting, tapping, or talkativeness. In adults, hyperactivity may manifest as extreme restlessness or wearing others out with their activity. Impulsivity refers to hasty actions that occur in the moment without forethought and that have high potential for harm to the individual (e.g., darting into the street without looking) (DSM V, APA, 2013).

Comorbidity is common in ADHD, with strong links to oppositional defiance disorder, learning disorders in children, major depressive disorder, anxiety disorders, social dysfunction, and substance abuse in adults. Academic issues surrounding ADHD in childhood are linked to a higher drop-out rate from secondary (high) school with fewer than 5% completing a university degree [97].

Conventional treatment options usually include, either in isolation or in combination, a pharmaceutical component, a behavioural component, and a psychosocial component. Pharmacotherapies. which inhibit the reuptake of noradrenaline and dopamine such as the psychostimulants methylphenidate and dextroamphetamine, and nonstimulating prefrontal cortex noradrenaline reuptake inhibitor atomoxetine, are the standard Western treatments for ADHD. Selective serotonin reuptake inhibitors (SSRIs) and other antidepressants are also used with varying degrees of success.

A third of ADHD patients who take stimulants for ADHD report significant adverse effects including anorexia, weight loss, abdominal pain, sleep disturbances, headaches, irritability, depressed mood, and appetite, with some reports of stimulant induced psychosis. Increasing apprehension regarding stimulant medication and the ramifications of its use in children has led to the investigation and acknowledgment of alternative therapeutic medications [94].

While more than 900 results can be found for the term “depression” in Pubmed, only 47 studies including just one systematic review can be found for the terms “oxidative stress” and ADHD [98]. When compared to oxidative stress, results from six studies with total 231 ADHD patients and 207 controls indicate that the association between ADHD and antioxidant status was not significant. However, results with markers of oxidative stress are controversial. Malondialdehyde (MDA), the marker of lipoperoxidation, was found increased in 20 adult patients and correlated with the score of hyperactivity [99], but in 30 children with ADHD, this parameter was not different from healthy controls [100]. In contrast to results of Oztop et al. [100], Essawy et al. [101] found higher level of MDA and decreased level of antioxidant element, zink, in children with ADHD.

Selek et al. [102] found increased level of NO which at low concentration exhibits important physiological functions in neurotransmitters release, memory, and learning [103], but at high concentration NO with superoxide can form very damaging oxidant, peroxynitrite ONOO^−^. At low SOD activity, which was found by Selek et al. [102], a redox imbalance and oxidative stress can be observed in adults with ADHD. However, in children, decreased activity of SOD was not observed [104]. Authors found altered activities also of other antioxidant enzymes, glutathione peroxidase, and nonsignificantly changed catalase in comparison to the controls. Our results found in 61 children with ADHD investigated in double-blind, randomized, and placebo controlled study suggest increased level of the marker of oxidative damage to DNA, 8-oxo-7,8-dihydroxyquanine (8-oxo-G) and decreased total antioxidant status in comparison to the controls [24]. We also investigated levels of neurotransmitters in urine. In ADHD children, adrenaline and noradrenaline concentrations positively correlated with plasma levels of oxidized glutathione and noradrenaline positively correlated with the degree of hyperactivity [105].

However, the determination of only one or two markers of oxidative stress cannot reflect the real redox state in the organism. Therefore, the evaluation of total oxidative status (TOS) and total antioxidant status (TAS) and their ratio as oxidative stress index (OSI) could be useful for identification of redox imbalance [106–108].

However, a small number of studies and their variety do not allow drawing definitive conclusions concerning involvement of oxidative stress in pathophysiology of ADHD.

### 5.1. Polyphenols in ADHD Treatment

In experimental conditions,* Ginkgo biloba* extract (EGb 761) was tested on synaptosomal fraction prepared from mice cerebral cortex. EGb 761 significantly increased uptake of serotonin. Similar effect was observed, when synaptosomes were prepared from the cortex of mice treated orally with EGb 761. These observations were found in an area of suspected deficit in people with ADHD [109]. In ADHD patients several polyphenolic compounds were tested for treatment [110]. The extract from* Ginkgo biloba* at daily dose 80–120 mg administered during 6 weeks to fifty children treated with methylphenidate had no benefits in double blind, randomized, and placebo controlled study [111]. However, in another study increased dosage with the maximum of 240 mg/day was administered to 20 children with ADHD in an open clinical pilot study over 3 to 5 weeks. Improvement of ADHD symptoms, as well as brain-electrical activity was observed [112].

St. John's wort from* Hypericum perforatum* (900 mg/day) was used for treatment of ADHD symptoms in a double blind, randomized, and placebo controlled study with 54 children. Positive results were observed after 8 weeks of treatment [113]. The effect of traditional Chinese medicine compound (*Ningdong*, NDG) at daily dose of 5 mg/kg was studied in 72 children with ADHD and compared with effects of methylphenidate (1 mg/kg) in a randomized double-blind trial. After 8 weeks of treatment NDG significantly reduced ADHD symptoms. The level of dopamin was not changed but serum level of homovanillic acid (a degrading product of catecholamine catabolism) increased [114].* Oroxylin A* is an O-methylated flavone, a chemical compound that can be found in the medicinal plant* Scutellaria baicalensis* and the* Oroxylum indicum* tree. It has demonstrated a dopamine but not noradrenaline, reuptake inhibitor activity. Its analogue, 5,7-dihydroxy-6-methoxy-4′-phenoxyflavone, showed the most remarkable inhibition of dopamine reuptake comparable to methylphenidate, but not modulation of GABA pathway in spontaneously hypertensive rat model of attention-deficit hyperactivity disorder [115, 116].

*Pycnogenol* (Horphag, Ltd), a standardized extract of French maritime pine bark* Pinus pinaster*, was also studied in relation to mental health, especially to ADHD. Pycnogenol is a defined mixture of polyphenols, mainly procyanidins, catechin, taxifolin, and a small amount of phenolic acids [117]. It exhibits a number of biological activities, especially antioxidant properties* in vitro* and many different biomodulating activities* in vivo* [118]. The exact mechanism by which Pycnogenol improves brain functions and mental health is not entirely clear yet. Several works on different levels (cell cultures, experimental animal models, and human studies) deal with effects of Pycnogenol on brain functions or mental health. The first condition for the positive effect of substances in the brain is the ability to cross the blood-brain barrier. Pycnogenol is able to cross blood brain barrier [119] as well as other cell membranes. Kurlbaum et al. [120] analysed the binding of constituents and the metabolite M1 (delta (3,4-dihydroxyphenyl)-gamma-valerolactone) of Pycnogenol that had been previously detected in plasma samples of human Pycnogenol consumers, to human erythrocytes. Authors found a transporter-mediated accumulation of the flavonoid metabolite, probably via GLUT-1 transporter. It was also found that Pycnogenol significantly increased the membrane fluidity predominantly at the membrane surface. Pycnogenol efficacy to modify effectively some membrane dependent processes is related not only to the chemical action of Pycnogenol but also to its ability to interact directly with cell membranes and/or penetrate the membrane thus inducing modification of the lipid bilayer and lipid-protein interactions [121]. The ability to modify membrane fluidity can be related to the pathology of psychiatric disorders through modification of adrenergic receptors [122]. Pycnogenol protected cultured SH-SY5Y neuroblastoma cells against acrolein-induced oxidative stress toxicity probably through its antioxidant properties and increased level of GSH [123]. The same cells were used in another experiment, in which Pycnogenol and extract from* Hypericum perforatum* (St. John's wort) were used as alternatives to the classical ADHD drugs. Pycnogenol exerted no significant effect on ATP level but increased cell survival at the concentrations 32.25 and 250 ng/mL [124].

Also results obtained from animal models support the positive effects of Pycnogenol on mental health. Increased oxidative stress is implicated in the pathogenesis of Parkinson disease in which dopaminergic neurons are intrinsically susceptible to oxidative stress. In Parkinson disease model mice treated with Pycnogenol (20 mg/kg) for 15 days decreased number of dopaminergic D2 receptors and increased levels of dopamin and its metabolites were observed [125]. Neuroprotective effect of Pycnogenol was observed by Scheff et al. [126] in a rat model after traumatic brain injury following increased oxidative stress, increased level of proinflammatory cytokines in cortex and hippocampus. In treated animals ameliorated level of protein carbonyls, lipid peroxides, protein nitrations, and proinflammatory cytokines were observed. In mentioned rat model the same group of authors also observed decreased level of thiobarbituric acid reactive substances (TBARS) in brain and injury-related declines in pre- and postsynaptic proteins after Pycnogenol treatment (1–10 mg/kg) [127].

Influence of Pycnogenol on cognitive functions and enhancement of “normal” mental performance was studied in 53 students in evaluation study. After 8 weeks of supplementation, attention, memory, executive functions, and mood rating were improved [128]. Influence of Pycnogenol on cognitive functions, attention, mental performance, and specific professional skills together with oxidative stress in healthy professionals was studied in 30 subjects and results were compared with comparable control group. After 12 weeks of Pycnogenol supplementation at the dose of 150 mg/day improved cognitive functions and oxidative stress parameters compared to the control group [129].

First case reports about positive effects following supplementation of ADHD; children with Pycnogenol were collected by Passwater [130]. Heimann [131] reported that coadministration of Pycnogenol and dextroamphetamine clearly improved symptoms of ADHD of a 10-year-old boy. Withdrawal of Pycnogenol while continuing dextroamphetamine treatment caused a relapse; reinstated Pycnogenol caused again the significant improvement. Positive experience with Pycnogenol was also reported by Hanley in her book “Attention Deficit Disorder” [132]. Masao published in Japan a success rate of 70% when treating 40 children with 1 mg/kg Pycnogenol [133]. An attempt to demonstrate reduction of ADHD symptoms in adults failed in a double-blind, placebo controlled, comparative study with 24 adults [134]. No significant differences were found between placebo, methylphenidate, and Pycnogenol groups. As the study could not show a difference between the active drug, methylphenidate, and placebo, the relevance of these results is questionable.

One randomized, double blind, and placebo controlled study examined the role of Pycnogenol in alleviating ADHD symptoms. 61 children with ICD-10 diagnoses of ADHD were enrolled to either Pycnogenol or placebo groups. Children in Pycnogenol group were administered Pycnogenol at the dose of 1 mg/kg/day for one month followed by 1 wash-out month. No serious side effects were reported. A significant reduction of symptoms was noted in the intervention group of the teacher-rated Child Attention Problems for hyperactivity and inattention, with symptoms returning to pretreatment levels after the wash-out period. Reduction of these symptoms was not observed in the placebo group. When rated by parents and teachers on Conners' rating scale, symptoms decreased slightly compared to the baseline and placebo but did not reach significance. Also positive effects were detected on visual-motor coordination and concentration tasks in intervention but not in the placebo group. The relatively small number of 44 patients treated with Pycnogenol and the short duration of the study limits the generalization of our findings [17]. In this study, also levels of catecholamines in urine were investigated. Patients suffering from ADHD had significantly higher levels of adrenaline and noradrenaline at the baseline compared to healthy age-matched controls. The concentration of noradrenaline in urine of patients with ADHD positively correlated with the score for inattention. Treatment with Pycnogenol resulted in significantly decreased dopamine levels, while adrenaline and noradrenaline showed only a trend toward reduced levels [105]. Parallelly, improvement of GSH/GSSG ratio was determined [135] as well as an increase of total antioxidant status and decrease of oxidative damage to DNA [24]. These results indicate that Pycnogenol can inhibit oxidative stress by normalizing catecholamine levels in children with ADHD, which may, in turn, reduce hyperactivity and increase attention [110]. After completion of the study, parents asked that their ADHD children continue the additional treatment with Pycnogenol. Mentioned studies indicate that Pycnogenol could become a promising additive and complementary supplement in ADHD treatment; however, more studies are needed to confirm this conclusion [136].

## 6. Schizophrenia

Schizophrenia is a devastating mental disorder, expressed in the form of abnormal mental functions and disturbed behaviour. It has a life-time prevalence of approximately 1% of the world's population [137]. Genetic and early environmental factors, as well as psychological and social processes, appear to be important contributory factors. Many possible combinations of symptoms have triggered debate about whether the diagnosis represents a single disorder or a number of separate syndromes.

Symptoms begin typically in young adulthood, and about 0.3–0.7% of people are affected during their lifetime. The disorder is thought to mainly affect the ability to think, but it also usually contributes to chronic problems with behavior and emotions. People with schizophrenia are likely to have additional comorbidity, including major depression and anxiety disorders. Social problems, such as long-term unemployment, poverty, and homelessness, are common. The average life expectancy of people with the disorder is 12 to 15 years less than those without schizophrenia. This is the result of increased physical health problems and a higher suicide rate (about 5%). The mainstay of treatment is antipsychotic medication, which primarily suppresses dopamine receptor activity. Some recreational and prescription drugs appear to cause or worsen symptoms.

It is assumed that increased oxidative stress may be relevant to the pathophysiology of schizophrenia [138]. Molecular mechanisms contributing to oxidative stress are very complex and not fully understood yet. Although oxidative stress may not be the main cause, oxidative damage to important biomolecules has been suggested to be a common pathogenic process contributing to deteriorating course and poor outcome [139, 140]. Brain has a high rate of oxidative metabolic activity (see chapter, Psychiatric disorders). Moreover, neurotransmitters (dopamine, adrenaline, and noradrenaline) present in excess in the brain can be autooxidized to form relatively large amount of hydrogene peroxide. Additionally, neuronal mitochondria can form excess of superoxide anion radical. Due to insufficient activity of Mn-superoxide dismutase (MnSOD) and low concentration of major free radical scavenger in brain, glutathione (GSH), mitochondria become damaged and dysfunctioned [141]. Glutathione and redox regulation have a critical role in myelination processes and white matter maturation in the prefrontal cortex of rodent and human, a mechanism potentially disrupted in schizophrenia [142]. However, data for the brain redox status are limited and contradictory in human. The majority of information for oxidative stress in schizophrenia is received predominantly from determination of markers in plasma/serum, blood cells, or urine, respectively. Reduced level of GSH was observed in plasma of patients with schizophrenia [143]. The lower level of another endogenous low-molecular weight antioxidant, uric acid, was found in plasma of schizophrenic patients [144]. The presence of this antioxidant in the CNS is limited by the blood-brain-barrier and is about ten times lower than in blood [145]. Concerning activities of antioxidant enzymes, such as superoxide dismutase, glutathione peroxidase, or catalase, controversial results in their activities (decreased, increased, and unchanged in comparison to healthy subjects) were observed in schizophrenic patients [146].

Similarly, contrasting results were observed in markers of lipid peroxidation (malondialdehyde, thiobarbituric acid reactive substances (TBARS), 4-hydroxynonenal, and isoprostanes) in patients with schizophrenia [147]. Meta-analysis of studies on MDA levels in schizophrenic patients showed very large heterogeneity of the results [148]. More accepted and more sensitive marker of nonenzymatic lipid peroxidation is F2-isoprostane, the product of peroxidation of arachidonic acid liberated from phospholipids [149]. This marker was found to be increased in patients with schizophrenia [150]. Also some other markers were investigated in schizophrenic patients. There were monitored markers of oxidative damage to proteins (protein carbonyls or 3-nitrotyrosine) [150], DNA such as 8-oxo-7,8-dihydro-2-deoxyguanosine, which was increased by 20% in 40 schizophrenic patients when compared to the controls [151], or leukocyte telomere length in 53 schizophrenic patients which was found to be gender dependent but not different from controls [152].

Oxidative stress is also related to apoptotic hypothesis of schizophrenia. Apoptosis (a programmed cell death) is a mechanism of cell death that operates in normal neurodevelopment and is increasingly recognized for its role in diverse neuropathological conditions. Activation of apoptosis can lead to rapid and complete elimination of neurons and glial cells in the CNS. In certain conditions, proapoptotic triggers can lead to sublethal and localized apoptotic activity that produces neuritic and synaptic loss without causing cell death. Neuropathology of schizophrenia includes reduced neuropil (especially synaptic elements) and limited and often layer-specific reduction of neurons suggesting progressive loss of cortical gray matter in first episode of psychosis, when antioxidant activity is low [153, 154]. Apoptotic mechanism that can influence synaptic connectivity and neuronal complexity seems to support the apoptotic hypothesis of schizophrenia connected also with oxidative stress [155].

Oxidative stress markers could be used to indicate the degree of severity of the disease in untreated schizophrenic patients and may be associated with the subtype of disorder [156].

### 6.1. Polyphenols in Schizophrenia

There are no studies yet, reporting the direct interference of polyphenols with pathophysiology or pathobiochemistry of schizophrenia in human. Understanding of the molecular foundations of schizophrenia pathophysiology would allow a targeted application of pharmacotherapy. However, this cannot be studied in human trials. Therefore, especially in chronic neurodegenerative and psychiatric disorders, the use of animal experiments is necessary. Conclusions of these experiments may then be more or less used for application in human biomedical field.

Preclinical studies suggest that the green tea extract with the main polyphenol* epigallocatechin-3-gallate (EGCG)* may possibly benefit patients with schizophrenia. Loftis et al. [157] were interested in whether EGCG at doses of 600 mg per day is a useful adjunct for maintenance treatment with antipsychotic medication in 34 patients in the double-blind and placebo controlled study. Authors have not found therapeutic effects of EGCG on psychotic symptoms in comparison to placebo. In schizophrenic patients only few works investigated the influence of polyphenols on side effects following antipsychotic treatment. Tardive dyskinesia (TD) is a serious adverse effect associated with the long-term administration of neuroleptics. The pathophysiology of antipsychotic treatment-induced TD is still unclear, although several reports assumed that free radicals may be involved [158]. Involvement of oxidative stress in the development of haloperidol-induced orofacial TD was confirmed by Bishnoi et al. [159]. Authors found that chronic administration of haloperidol increased vacuous chewing movements, tongue protrusions, facial jerking, and also oxidative damage in all major regions of rat brain. These changes were dose-dependently inhibited by curcumin. Authors point to curcumin as a possible therapeutic option to treat this hyperkinetic movement. Similarly, in experimental conditions flavonoid quercetin (3,5,7,3′,4′-pentahydroxyflavone) reverses haloperidol-induced extrapyramidal side effects, catalepsy, usually associated with catatonic schizophrenia. It is a physical condition, characterized by suspension of sensation, muscular rigidity, fixity of posture, and often loss of contact with surroundings [160]. Besides this, quercetin and also resveratrol (3′,4′,5′-trihydroxystilbene) reduced lipid peroxidation in human plasma caused by a first-generation antipsychotics, haloperidol in* ex vivo* experiments. The amisulpride, the second-generation of antipsychotic drugs did not influence the level of lipid peroxidation biomarker TBARS in comparison to the controls [161].

Flavonoid epicatechin, present as a major component in green tea, inhibits lipid peroxidation in human plasma caused by haloperidol in experiment* ex vivo* [162].

Plasma lipid peroxidation induced by atypical antipsychotic drug ziprasidone was also inhibited by polyphenols from berries isolated from* Aronia melanocarpa* in* ex vivo* experiments [163]. However, results of experiment* ex vivo* should be read and interpreted with caution, because polyphenols passing through the GIT are metabolized to derivatives and, therefore, the effect on lipid peroxidation* ex vivo* may not be identical to the effect of* in vivo*.

Extract from* Ginkgo biloba (EGb-761)*, which components are mostly lipophilic, crosses the blood-brain barrier and protects the brain against damaging effect of oxidative stress. In the study by Zhang et al. [164], 157 patients suffering from schizophrenia were included in the double-blind and placebo controlled study. Patients in EGb-761 group were administered daily dose of 240 mg EGb-761 for 12 weeks. Significant improvement of TD symptoms in schizophrenic patients was observed in EGb-761 group in comparison to placebo. The improvement may be mediated through the well-known antioxidant activities of this extract.

Genistein, a polyphenol belonging to phytoestrogens together with amino acid leucine, is able to potentiate the haloperidol-induced catalepsy in rats compared with the haloperidol treated group and reduced the number of fights and increased latency to fights in foot shock-induced aggression [165].

Since not all polyphenols are able to pass through the blood-brain barrier, it is necessary to look for new therapeutic approaches. One of the new approaches is the use of exosomes. Exosomes are small (30–150 nm) extracellular cell membrane-derived vesicles that are present in many and perhaps all biological fluids, including blood and urine. Exosomes are either released from the cells when multivesical bodies fuse with the plasma membrane or released directly from the plasma membrane. It is becoming increasingly clear that exosomes have specialized functions and play a key role in, for example, coagulation, intercellular signaling, and waste management. Exosomes' simple structure and abilities to be incorporated into plasma membrane and to cross the blood-brain barrier allow them to be utilized as drug delivery vehicles (in our case polyphenols) or genetic elements in the treatment of immune, psychiatric, and neurologic disorders [166].

Several questions remain open for the role of oxidative stress in schizophrenia. Antipsychotic drugs have been suspected to generate increased ROS resulting in increased oxidative stress. What kind of antipsychotic drugs is involved in oxidative stress? What are the symptom domains associated with the oxidative stress? Is the oxidative stress an attribute of early or chronic stages of the disease? What is the role of current treatment on oxidative stress? The answer to these questions and explanation of the participation of oxidative stress in pathology of schizophrenia need further validation [167].

## 7. Conclusions

A large number of studies have focused on investigation of effects of natural polyphenols in mental disorders, but their use in clinical practice is still a long way off [168]. There might be several reasons for such a slow and ineffective research.

(1) There are no sufficient sophisticated analytical methods for determination of levels of polyphenolic compounds and their metabolites in brain, (2) it is very difficult to find a suitable animal model that would mimic the exact status of human mental disorder, (3) isolated studies of interorgan actions and reactions between brain and peripheral organs cannot give the complex view, (4) application of information obtained from* in vitro* or* ex vivo* experiments into* in vivo* conditions of the complex nervous system is complicated by the biotransformation of original polyphenols to entirely different metabolites, and (5) antipsychotic effects of polyphenols have not been sufficiently validated in clinical practice yet.

Due to the enormous complexity of the human brain, the exact pathophysiology of psychiatric disorders is not known yet and the understanding of these complex relations needs to collect huge amount of data on all levels of research, experimental and human.

Identification of the exact mechanism of pathological components of mental disorders on molecular level can lead to the development of effective treatments. Polyphenols in the diet have the potential to become medicaments in the field of mental health after a thorough study of their mechanism of action. Members of the International Society for Nutritional Psychiatry Research advocated recognition of diet and nutrition as central determinants of both, physical and mental health [169].
